# Theoretical Studies in Molecular Dynamics and DFT of the Interaction between Imidacloprid in Polyethylene and Polypropylene Surfaces

**DOI:** 10.1021/acsomega.5c01415

**Published:** 2025-04-24

**Authors:** Leonardo Paes da Silva, Cristiani Lopes Capistrano Gonçalves de Oliveira, Adriana Nunes Correia, Pedro de Lima Neto, Norberto de Kássio Vieira Monteiro

**Affiliations:** † Departamento de Química Analítica e Físico-Química, Centro de Ciências, Universidade Federal do Ceará, Campus do Pici Bloco 940, Fortaleza, Ceará 60440-900, Brasil; ‡ Departamento de Farmácia, Universidade Federal do Ceará, Campus do Porangabussu Fortaleza, Ceará 60430-372, Brazil

## Abstract

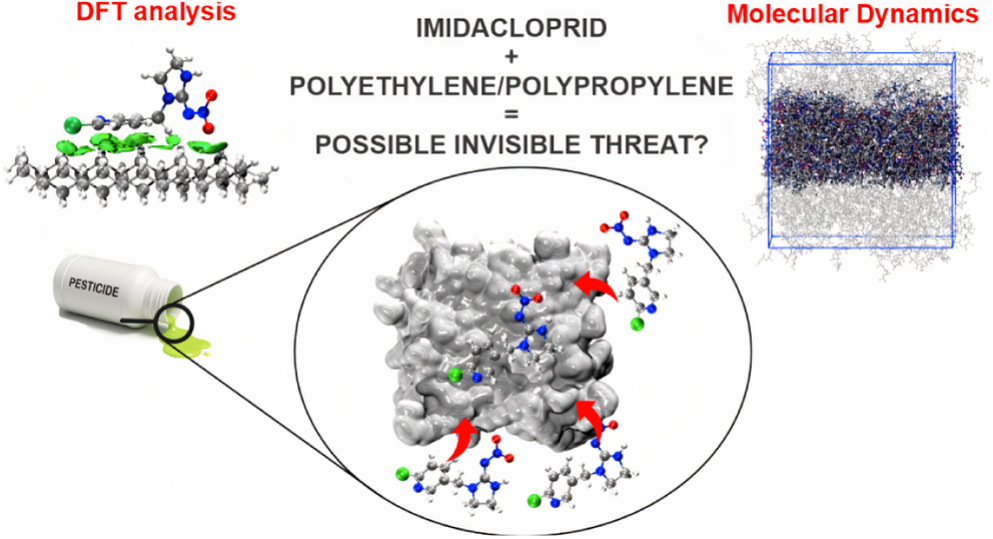

Pesticides are chemical substances that are often used in agriculture to correct soil deficiencies, control pests, and eradicate destructive plants. However, it is imperative to assess their effectiveness to avoid potential harm to human health. In addition, microplastics (MP) have been the subject of research into their spread from marine and agricultural environments. Considering the possibility of contact between pesticides and microplastics, with the subsequent possibility of them acting as vectors of dispersion through adsorption between the two, it is imperative to evaluate the effectiveness of pesticides in order to avoid potential harm to human health. The current study used computational calculations to analyze the possible interactions between polyethylene (PE) and polypropylene (PP) microplastics with the pesticide imidacloprid (IMI), which is used in the cultivation of bananas, one of the most widely grown fruits in the world. Molecular dynamics (MD) and density functional theory (DFT) calculations indicated favorable adsorption energies for the interaction of the two microplastics. The results obtained by applying MD and DFT indicate that the nature of the IMI–MP interaction is van der Waals. Consequently, the theoretical approaches suggest that the pesticide under study has a strong propensity to interact with PE and PP, providing a significant incentive for future experimental investigations in this area.

## 1 Introduction

The basis of society is agriculture, which needs large-scale food production and is a crucial pillar of the global economy.^1−3^ The growing population demand has intensified the need for agriculture to keep up with the population explosion, leading to a greater demand for fertile soils and planting efficiency in agricultural areas.^2,4,5^ However, the excessive, continuous, and improper use of soil can cause soil impoverishment and reduce soil efficiency in addition to persistent pest problems. Agrochemicals aim to rectify soil deficiencies, control pests, and eliminate crop destruction. To enhance agricultural production, agrochemicals aim to rectify soil deficiencies, control pests, and eliminate crop destruction.^6−8^ A pesticide is a substance to prevent, destroy, or manage pests, including human and animal disease transmission agents. These unwanted species of plants or animals can cause damage. In addition, their application is common during the production, processing, storage, transportation or distribution of food, agricultural products, wood, and derived products, as well as for controlling insects, arachnids, and other pests that affect the body of farm animals.^9^ However, it is essential to consider the practical aspects of pest control, considering their probable harmful impacts on human health, whether chronic or acute. The assessment and categorization of the potential environmental risks of a pesticide are based on physicochemical, toxicological, and ecotoxicological research.^10−12^

There are four primary categories of pesticides, including insecticides, herbicides, fungicides, and rodenticides, each with a specific target pest and potential toxic effects. The mode of exposure is crucial in comprehending the effects of pesticides, as exposure can occur through various routes, such as ingestion, inhalation, and skin contact, which determine the severity of toxic impacts.^13−15^ It is also important to understand that pesticides can produce acute and chronic toxic effects. Cute effects manifest shortly after exposure and can include symptoms, such as headaches, waves of nausea, and vomiting. On the other hand, chronic effects can occur after prolonged or repeated exposure and may include conditions like cancer, reproductive disorders, and neurotoxicity.^16^

Pesticides have been demonstrated to contaminate groundwater through various mechanisms, including soil penetration and the use of surface water for irrigation. These practices enable the migration of pollutants from agricultural regions to groundwater during substantial precipitation, such as heavy rainfall or flooding. The propensity for this phenomenon is amplified under conditions of excessive pesticide application or when the soil is saturated, thereby facilitating the unobstructed movement of pesticides. The application of pesticides in these areas can be effectively washed away by rainwater, leading to the contamination of nearby bodies of water such as rivers and lakes. This infiltration process eventually results in the migration of pesticides into the groundwater. Accidental spills or leaks of pesticides during storage, transportation, or application can also result in groundwater contamination.^17,18^

Additionally, the injection of pesticides into drainage wells intended to remove excess water from the soil may cause groundwater contamination. Once pesticides reach groundwater, they can persist for extended periods, depending on their chemical properties and environmental conditions. Some pesticides can remain in groundwater for years, and their concentrations can increase over time, posing a significant risk to human health.^18−21^

Bananas are among the most widely consumed fruits globally, with Brazil having the highest consumption rate. In 2020, the banana industry generated approximately 1.6 billion dollars in Brazil due to the favorable planting conditions in the country, with an estimated 450,000 ha devoted to banana cultivation.^22−24^ As with any crop, effective pest control is crucial for maximizing the production efficiency and profitability in banana farming. Pests can severely affect the quality and quantity of the production. The use of pesticides has been proposed as a solution to preventing and controlling these problems. However, there is growing concern about the potential health and environmental risks associated with the misuse of pesticides. In Brazil, the use of pesticides is regulated by the ANVISA (National Health Surveillance Agency), MAPA (Ministry of Agriculture and Livestock), and IBAMA (Brazilian Institute of Environment and Natural Resources). A list of the most commonly used pesticides for banana crops, as approved by these regulatory agencies: Azoxystrobin: fungicide; Bifenthrin: insecticide, formicide, and acaricide (versatile, with three actions); Diurom: herbicide; Epoxiconazole: fungicide; Glyphosate: herbicide (the most consumed in the world); Imidacloprid: insecticide (acts on the cells of the central nervous system of insects such as bees, reducing pollination power); Mancozeb: fungicide and acaricide (versatile, with three actions); Pyraclostrobin: fungicide; Tebuconazole: fungicide.^25^

Plastic materials are widely used as containers for various substances including pesticides. Plastics have become a ubiquitous and persistent environmental pollutant, with microplastics (MPs) being a particular concern due to their widespread presence in marine and freshwater environments.^26^ Despite the benefits of plastics, they are increasingly recognized as materials responsible for harmful environmental effects, particularly due to the presence of MPs.^27^ While pollution with plastics and microplastics in aquatic environments has received significant attention, the issue of soil pollution with plastics remains relatively unexplored.^28^ The use and loss of plastic products contribute to the increasing amount of microplastics entering the environment, posing threats to ecological and human health.^29^ The research on environmental MPs aims to stimulate further investigation and calls for papers to address this critical issue.^30^

Microplastics are chemically polymers since they consist of polyethylene (PE), polypropylene (PP), polyamide (PA), polyvinylene (PV), polypropylene (PP), polystyrene (PS), chloride (PVC), and polyethylene terephthalate (PET).^31^ Based on the provided references, it is evident that polyethylene (PE) and polypropylene (PP) MPs have been extensively studied in various environmental contexts. Studies have reported the presence of these microplastics in diverse environments, including marine, freshwater, and terrestrial systems.^32−38^ Furthermore, the distribution patterns of microplastics in urban freshwaters have revealed the common occurrence of PE and PP MPs.^39^ Additionally, PE and PP microplastics have been identified in South Maldives’ fishes, emphasizing their prevalence in marine environments.^40^ Moreover, the distribution characteristics of microplastics in the soil of mangrove restoration wetlands have indicated the presence of PE and PP as the main soil MP polymers.^41^

The adsorption of pollutants on MPs, such as PE and PP, has been the subject of extensive research. Studies have shown that microplastics possess high adsorption capacities for various pollutants in aquatic and soil environments.^42^ The adsorption process is influenced by particle size, surface area, and the nature of the MP material.^43^ The association among PE, PP, and contaminants extends to their role in the transport and bioavailability of chemical contaminants. For instance, PE has established an equilibrium with the surrounding water, influencing the diffusion and partitioning of contaminants, such as polycyclic aromatic hydrocarbons.^44^

Additionally, buoyant PE and PP MPs have been documented in hydrophobic contaminant-rich environments, indicating their potential to transport hydrophobic contaminants.^45^ The adsorption/desorption kinetics of contaminants may differ depending on the type of MPs, with PE exhibiting higher adsorption than other types of microplastics.^46^ Additionally, the adsorption and desorption processes of specific contaminants, such as triclosan, on biodegradable polyhydroxybutyrate microplastics have been investigated, providing insights into the interactions between contaminants and different types of MPs.^47^ The interactions between contaminants, PE, and PP MPs have been studied in controlled laboratory conditions, elucidating the adsorption behavior of specific contaminants such as chromium and cadmium.^48,49^ Studies have investigated various pesticides’ adsorption mechanisms and behaviors on these MPs, shedding light on their potential environmental impact. For instance, Mo and coworkers^50^ focused on the adsorption behavior of pesticides, such as carbofuran, on PE and PP MPs, utilizing density functional theory (DFT) calculations to elucidate the adsorption mechanisms and particle size effects. A study by Li and coworkers^51^ investigated the adsorption of three pesticides on PE MP in aqueous solutions, providing insights into the kinetics, isotherms, thermodynamics, and molecular dynamics simulations of the adsorption process. Liu and coworkers^52^ investigate how ultraviolet (UV) radiation-induced photoaging affects the adsorption of the neonicotinoid insecticide imidacloprid (IMI) on two types of polar microplastics (MPs): polyamide (PA) and polylactic acid (PLA). The researchers observed significant changes in the surface morphology and chemical properties of both MPs after UV exposure. Specifically, photoaged PA MPs exhibited a disruption of C–N bonds leading to the formation of additional carbonyl groups, while aged PLA MPs showed degradation of oxygen-containing functional groups, resulting in smaller molecular fragments. These alterations influenced the adsorption capacities of the MPs: a 19.2% decrease for PA MPs and a 41.2% increase for PLA MPs postphotoaging. The primary adsorption mechanisms identified include electrostatic interactions, hydrogen bonding, van der Waals forces, and polar–polar interactions. Environmental factors such as higher pH levels and lower ionic strength were found to enhance IMI adsorption by affecting the surface charge distribution of the MPs. Understanding the adsorption behavior of pesticides on microplastics is crucial due to the potential role of MPs as vectors for transporting organic pollutants in aquatic environments. Computational chemistry methods, such as density functional theory (DFT) calculations and molecular dynamics (MD) simulations, have proven to be instrumental in elucidating the interactions between pesticides and MPs at the molecular level.

Therefore, given the possibility that plastic materials could carry pesticides into water currents and contaminate the population, this study selected PE and PP MPs to investigate their adsorption mechanism with the pesticide imidacloprid (IMI) using molecular dynamics (MD) and density functional theory (DFT). The computational investigation of pesticide adsorption on PE and PP MPs will provide valuable insights into the interactions between MPs and the pesticide, with implications for understanding environmental pollution, water quality, and human health.

## 2 Computational Details

### 2.1 MD Simulations

Four boxes with dimensions of 8 nm × 8 nm × 4 nm were constructed for the molecular dynamics simulation, and PE and PP molecules were inserted separately into each one. The PE and PP boxes were then equilibrated in the NVT ensemble for 100 ns. Subsequently, the boxes were expanded to a maximum size of 8 × 8 × 8 nm. They were filled with water in the TIP3P solvation model^53^ to represent imidacloprid in a dilute aqueous solution, adding an imidacloprid molecule. The second type of system was built to represent a concentrated solution of imidacloprid (pure solution). The number of imidacloprid molecules was added according to the average concentration of the products used, between 1400 and 1540 g L^–1^. Therefore, the system was filled with 420 molecules of imidacloprid, considering the volume of the simulation box. In this way, two PE systems were set up with diluted and pure imidacloprid and two PP systems with diluted and pure imidacloprid. After constructing the two-phase systems, the diluted and pure systems box went through two energy minimization steps (steep descent and conjugate gradient), 3 ns NVT equilibrium step with a V-rescale thermostat,^54^ 3 ns NPT equilibrium step with a Parrinello–Rahman barostat,^55^ and a production step under 200 ns NPT conditions in 298 K, at 1 bar. The Leap–Frog algorithm^56^ was applied to integrate the motion equation with a time step of 2.0 fs. The long-range interactions were modeled using particle-mesh Ewald sum (PME)^57^ with a cutoff of 1.2 nm. The LINCS algorithm^58^ was utilized to restrict hydrogen bonds. The MP molecules were represented by 25 monomers of their respective polymers and parametrized on the Acpype server.^59^ The force field used was that of amberff14sb_parmbsc1.

### 2.2 Conformation Analysis for Quantum Studies

**Figure 1:**
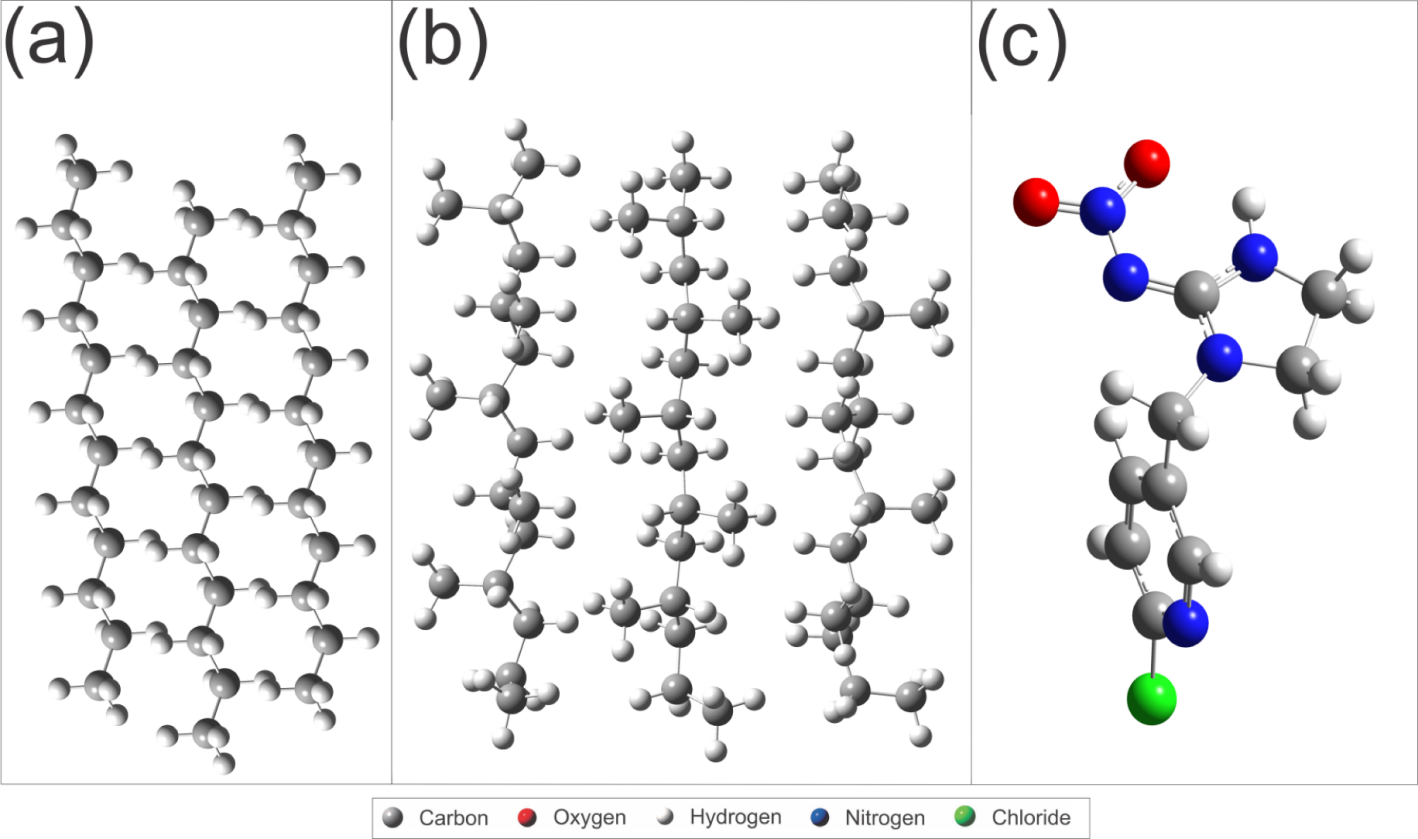
Geometrically optimized molecules of polyethylene surface (PE) (a), polypropylene surface (PP) (b), and IMI (c).

Initially, the PE and PP MPs surfaces were built to be sufficiently large to allocate the pesticide molecules. The PE and PP MP surfaces were constructed from three chains with 12-carbons length^60^ (Figure 1a,b). The first stage consisted of a geometric optimization of the PE and PP surfaces at the GFN2-xTB level,^61^ to research and compare a series of possible surface conformations. After optimization, an energy classification is carried out and the IMI–MP system with the lowest possible energy is selected for further study. Two thousand conformational frames were obtained from MD simulations for each pesticide MPs surface system studied. Using the semiempirical algorithm, all frames were geometrically optimized. Energy classification was performed using CREST 3.0 software^62^ with the ensemble’s NCI algorithm.

### 2.3 Density Functional Theory (DFT)

After obtaining the most stable conformations, DFT-level calculations were performed to obtain the adsorption energies between the pesticide and MP surface using Orca 5.0.4.^63^ The functional level used was B3LYP^64−66^ combined with the def2-SVP^67^ basis set in the solvation model conductor-like polarizable continuum model (CPCM),^68,69^ combined with solvation model based on density (SMD) solvation model in water. This combination was used because the CPCM model calculates the electrostatic contribution to solvation but does not account for the nonelectrostatic contributions, which are corrected by the SMD,^70^ which also empirically includes cavity effects. The geometric counter poise correction (gCP),^71^ implemented in the ORCA software, was used to correct the basis set superposition error (BSSE). The D4 empirical dispersion correction^72,73^ choice is due to the short- and long-range interactions, which includes a dispersion term, attempting to reproduce the Van der Waals effects. The adsorption energy between the pesticide and the MP was obtained through single-point calculations of electronic energy for each species individually and calculated using eq 1:
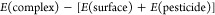
1

*E*_complex_ is the electronic energy of the IMI–MP (PE or PP) complex; *E*_surface_ and *E*_pesticide_ are the individual electronic energies of the PE or PP MP surface and pesticide studied, respectively. The energy decomposition analysis is based on the force field (EDA-FF).^74^

### 2.4 Weak Interactions Analysis

The independent gradient model Hirshfeld based on the Hirshfeld partition of molecular density (IGMH)^75^ calculations were utilized to analyze noncovalent interactions at the molecular level of pesticide–MP complexes. eq 2 is used to obtain the IGM function (δ*g*), ∇ρ(*r*) which is the gradient of charge density, and ρ(*r*) is the electron density. The reduced density gradient (RDG) versus λ_2_ρ(*r*) distribution shows different noncovalent interactions in values (λ_2_ρ(*r*)<0) that are strongly attractive, like hydrogen bonds; in values (λ_2_ρ(*r*)≈0), van der Waals interactions and repulsive interactions, and in values (λ_2_ρ(*r*)>0) a repulsive interaction such as steric hindrance.^76^

The function represents the gradient of the electronic density between the atoms. The function corresponds to *g*(*r*) as a type of gradient obtained by summing the absolute values of the density gradients of the atoms. The difference between these functions is expressed by
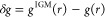
2

Defined as δ*g*, this value is different from zero in the region between the nuclei. In the context of fragment analysis, the region of interest is called fragment 1 for the pesticide and fragment 2 for the MP. Considering the function in real space, the interaction region between these fragments is identified within a fragment in {*A*} and can be determined from eqs 2–4.
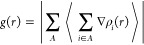
3
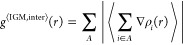
4
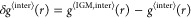
5

In the context of this study, the std parameter denotes the standard deviation of the electronic density along the dynamic trajectory. This value is calculated according to eq 5, in which “*n*” represents the total number of frames in the trajectory and “ρ(*r*)” indicates the electronic density associated with the geometry of the *i*th frame. In this way, “std” provides a quantitative measure of the fluctuations in the electronic distribution during the dynamic evolution of the system.
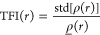
6

At the same time, the TFI is evaluated based on the variations in the color of the analyzed regions. In particular, the blue areas correspond to regions with high structural stability, while the green regions indicate interactions of intermediate intensity. On the other hand, red areas signal regions where interactions are more susceptible to modifications or distortions caused by thermal movements. This visual differentiation makes it easier to identify points in the system that may be more reactive or unstable, contributing to a detailed qualitative analysis of the molecular dynamics. The IGMH is defined as
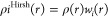
7being *w*_*i*_(*r*), Hirshfeld partition:
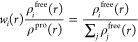
8

The AIGM, IGMH, AIM, EDA-FF, and DOS data were obtained by the MULTIWFN 3.8 software^77^ and visualized by the VMD software.^78^

## 3 Results and Discussion

### 3.1 Molecular Dynamics

#### 3.1.1 RDF

**Figure 2:**
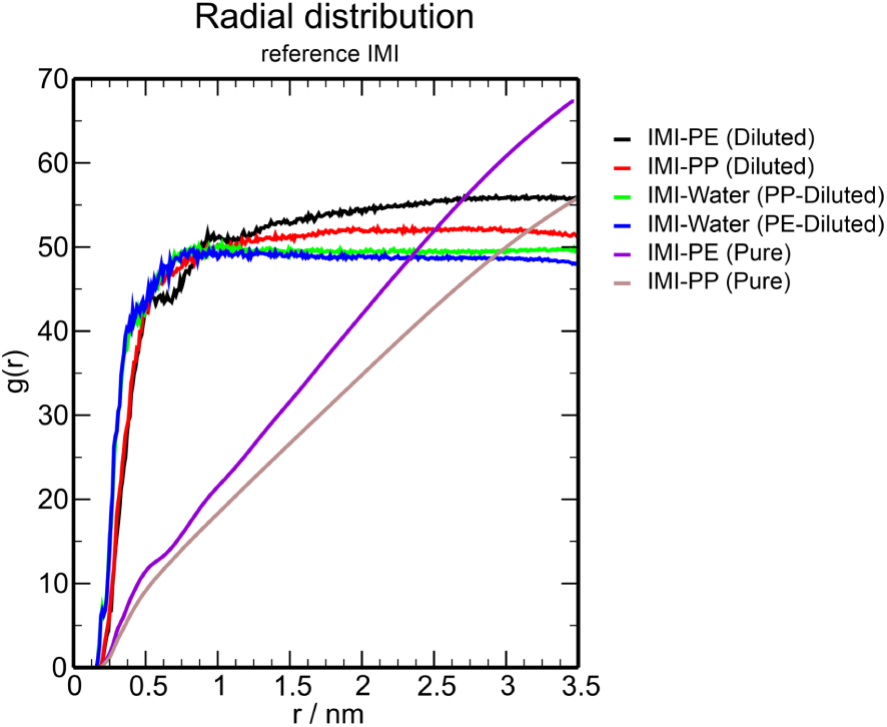
Radial distribution function plots for the IMI molecule.

The radial distribution function (RDF) indicator was used to determine how the imidacloprid species are distributed on the MP surface as a distance function and to analyze how the water molecules solvate the pesticide molecule. Figure 2 shows that IMI is more likely to interact with PE and PP surfaces than water in their respective systems in a dilute solution. However, when comparing diluted systems, IMI showed a similar *g*(*r*) at short distances for PE and PP (Figure 2) and a slight favoring for PE at longer distances. However, the distribution profile of IMI with water in the diluted systems was very similar, with values lower than 48. In addition, in the pure IMI solution, the graph indicates a greater distribution of pesticide species on the PE surface than on the PP surface, comparing them at equivalent distances.

#### 3.1.2 SASA

**Figure 3:**
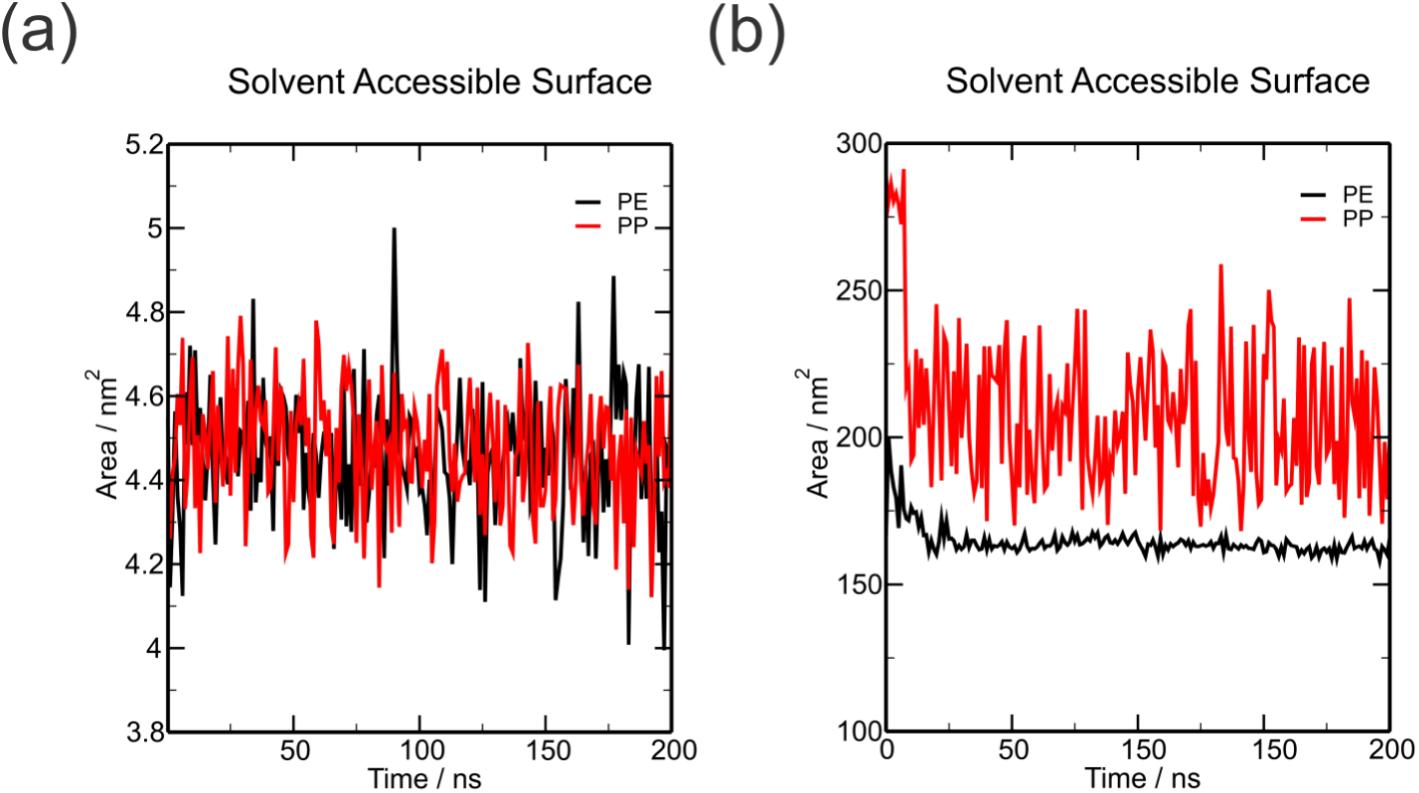
Time evolution of the solvent accessible surface area (SASA) in dilute (a) and pure (b) systems.

The solvent accessible surface area (SASA) index was obtained to check the area of IMI available to the solvent; therefore, the higher its index, the greater the contact with the available solvent. Therefore, in the system used to study the interaction of IMI on the MP surface, a lower SASA index means that the area available to the solvent has been reduced due to adsorption on the MP surface. The SASA indices for PE and PP were very similar in the diluted system since the system is represented by one molecule of imidacloprid, and water solvation will occur very similarly (Figure 3). However, in the pure IMI system, there was a significant variation in the surface area available in the PE system being smaller than the area available in PP. In addition, the SASA index in PE was more stabilized. At the same time, in the PP system, there were greater variations, indicating that in pure systems, imidacloprid in PE has a behavior that remains compact about its structure, while in PP, there were greater variations in the deformation of its structure, which were from 150 to 250 nm^2^ (Figure 3), indicating that interactions with PP led to noncompact aggregations on the surface and between imidacloprid molecules.

#### 3.1.3 Radius of Gyration (ROG)

**Figure 4:**
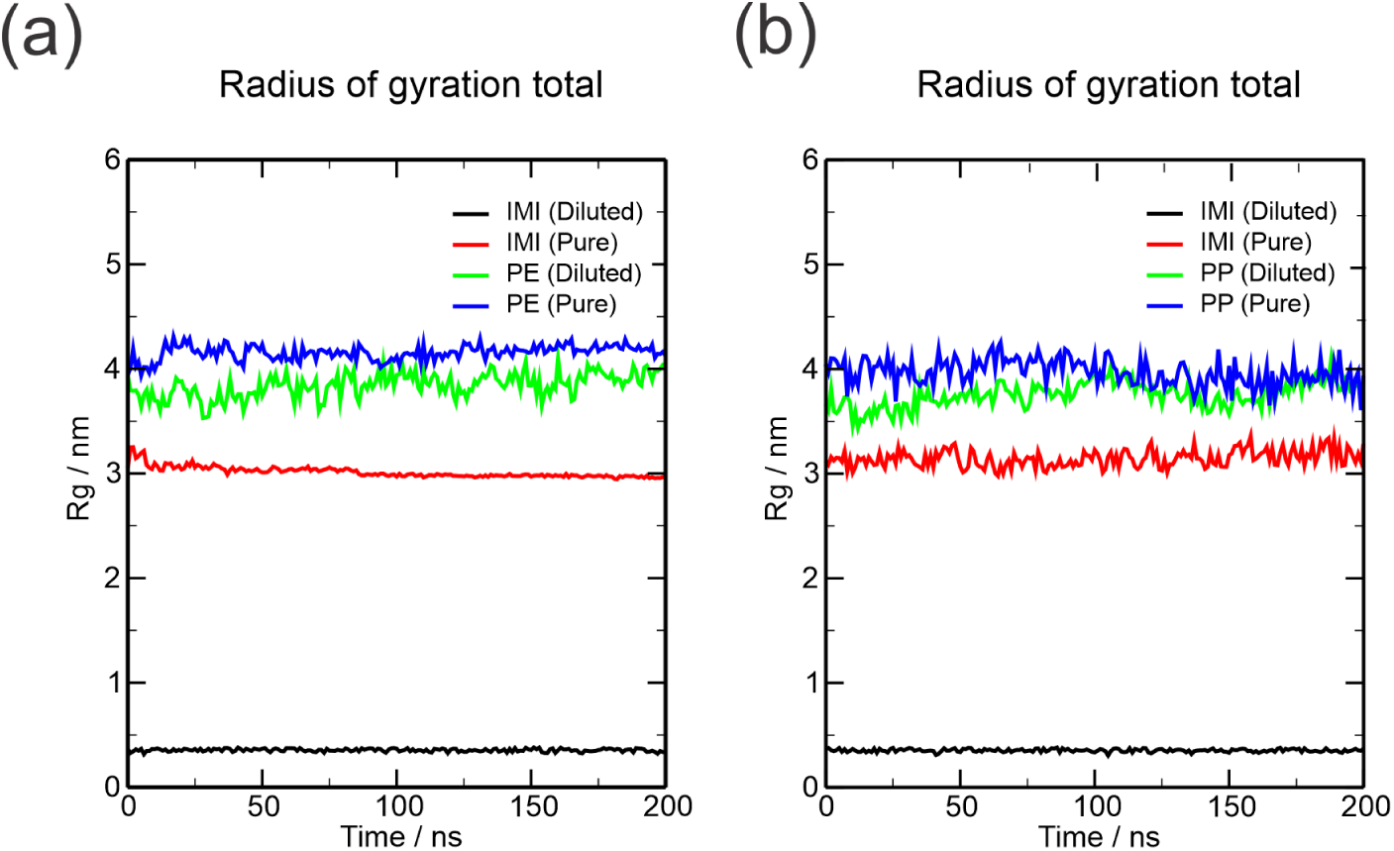
Time evolution of the radius of gyration in surface PE (a) and PP (b).

The ROG index also studies the compactness of the species in their respective systems. Looking at Figure 4, the ROG of the IMI species on PE in a diluted system remains stable at around 0.4 nm along the trajectory. The ROG of IMI on PE in a pure system is observed in the 3 nm range and remains stable. In the analysis of the PE surface, the index was lower in the diluted system than in the pure system, indicating that there is less surface compaction in the smaller system, which can be explained by the interactions with many more imidacloprid molecules, thus reducing the interactions between the PE molecules. The PP system showed similarities to that of the PE system. Still, the ROG of the pure IMI species showed greater variation, which corroborates the RDF and SASA data, indicating less stable compaction in a pure system.

#### 3.1.4 IPE

**Figure 5:**
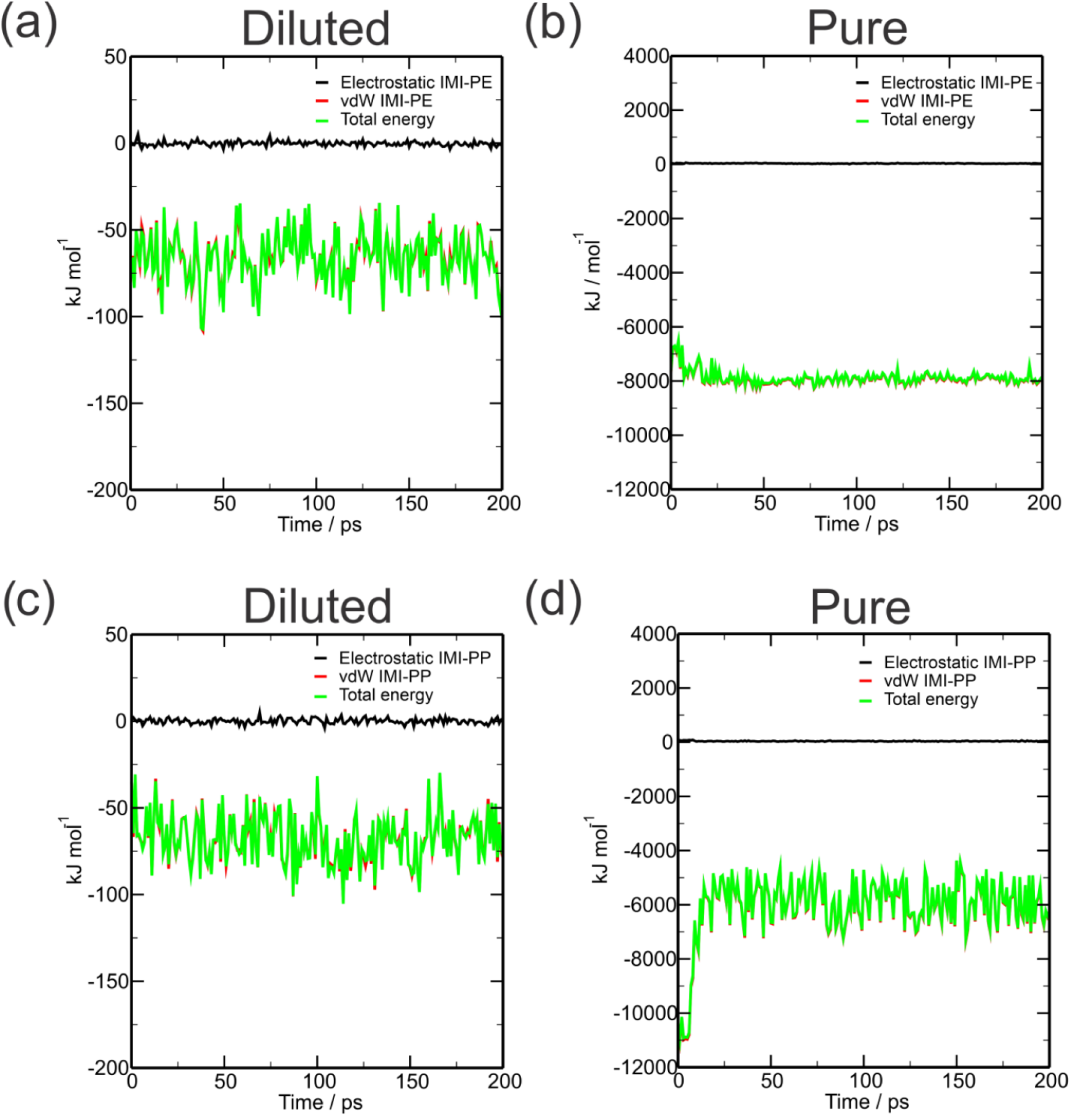
Time evolutions of the MPs–benzene interaction energy in systems dilute (a) and pure (b) in PE; systems dilute (c) and pure (d) in PP.

Figure 5a–d shows the interaction potential energy (IPE) between imidacloprid and microplastics. This calculation is obtained by the sum of short-range of van der Waals and electrostatic energies. In the diluted system, the pesticide obtained an average IPE interaction energy value of −66.28and −67.58 kJ mol^–1^ on the PE and PP surfaces, respectively (Figure 5 a–c), with values ranging between −34.71 kJ and −107.28 kJ mol^–1^. In the IMI-pure system, the interaction with PP obtained an average of −6035.17 kJ mol^–1^, while with PE, the average value was −7865.32 kJ mol^–1^ (Figure 5 b–d). The similar IPE energy profile in the diluted system corroborates the other data, suggesting that the imidacloprid molecule has similar attractiveness in the two MPs. Furthermore, the high magnitude of the adsorption values in pure systems is justified by the number of IMI in the system (420) interacting simultaneously with the surface, making the system denser due to the greater number of molecules. The periodic boundary conditions (PBC) used in the molecular dynamics also take into account the interactions between the IMI molecules in the upper region and the replicated MP molecules on the lower surface. Therefore, the IMI–MP interaction was more favorable, with average values extremely close to those of PE and PP in the diluted system, where only one IMI molecule is considered in a solvated environment. Favorable values for the interaction of imidacloprid with PE in the pure system can be explained by the larger contact area with the MP since PE has a higher average specific mass than PP, thus having a greater possibility of interacting with more PE molecules, corroborating the data from RDF, SASA, and ROG. Thus, the interaction energy of imidacloprid on the microplastic surface was governed by Lennard–Jones potential forces, which is a fundamental factor for the forces contributing to the adsorption of the pesticide to be van der Waals forces, given that the MP surface has no significant polarity.

### 3.2 Weak Forces Study

#### 3.2.1 AIGM Study

**Figure 6:**
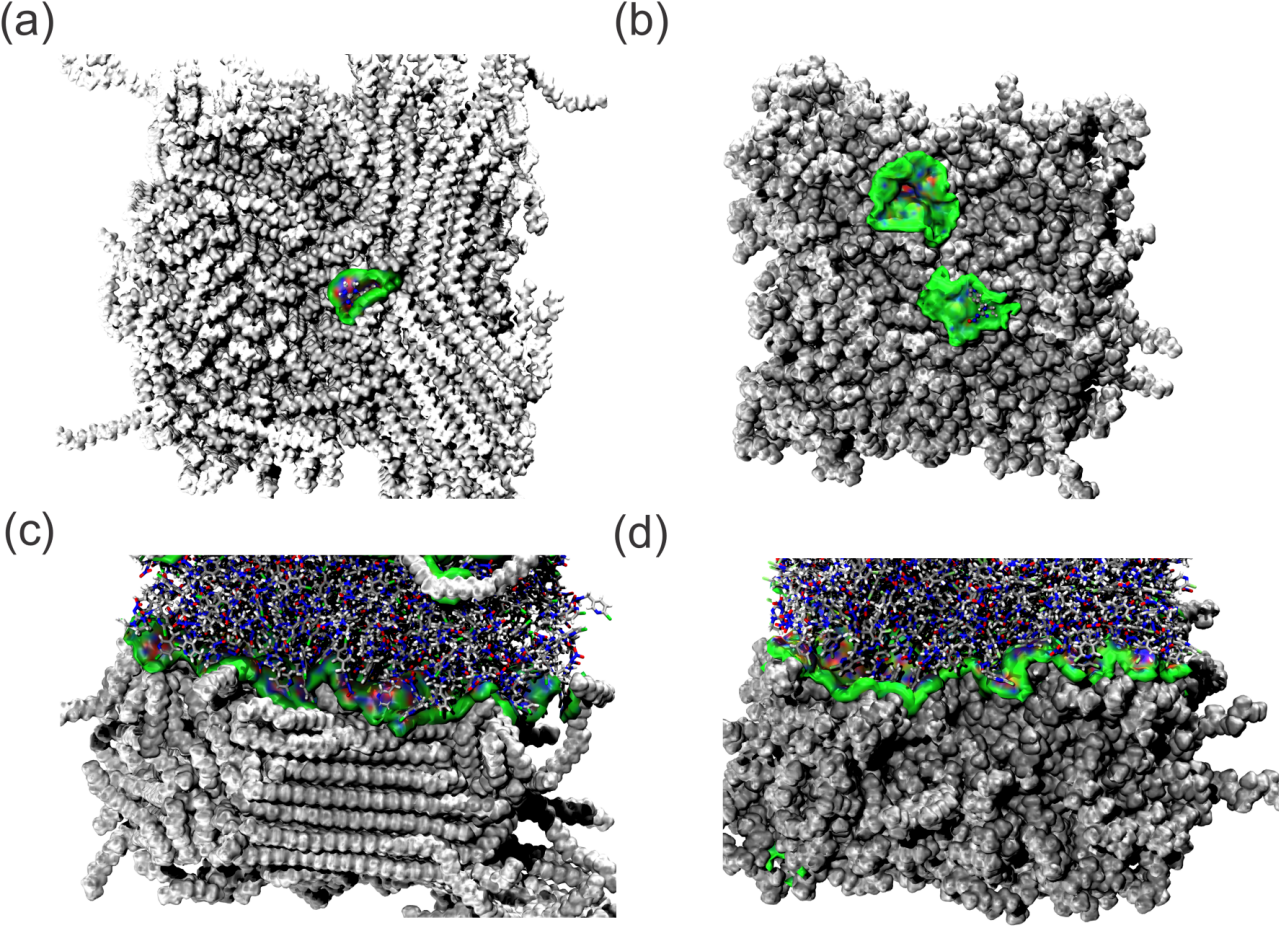
aIGM isosurface map of IMI–MPs interactions of the (a) PE diluted, (b) PP diluted, (c) PE pure, and (d) PP pure systems.

The IGM data are obtained from the 100 ns dynamic result with the MP atoms frozen, verifying only the IMI–MP interactions of the MD simulation process. This approach is evidenced by the strength of intermolecular interactions, where strong, attractive interactions, such as hydrogen bonds, are colored blue, medium-strength interactions, such as van der Waals forces, are colored green, and repulsive interactions are colored red. Figure 6a–d shows the nature of the intermolecular interaction between the IMI and MPs, confirming that short-range van der Waals forces governed this interaction throughout the simulation. It was also observed that there was a larger region of IMI interaction on the PP surface in the diluted system than on the PE surface.

#### 3.2.2 Thermal Fluctuations Index (TFI)

**Figure 7:**
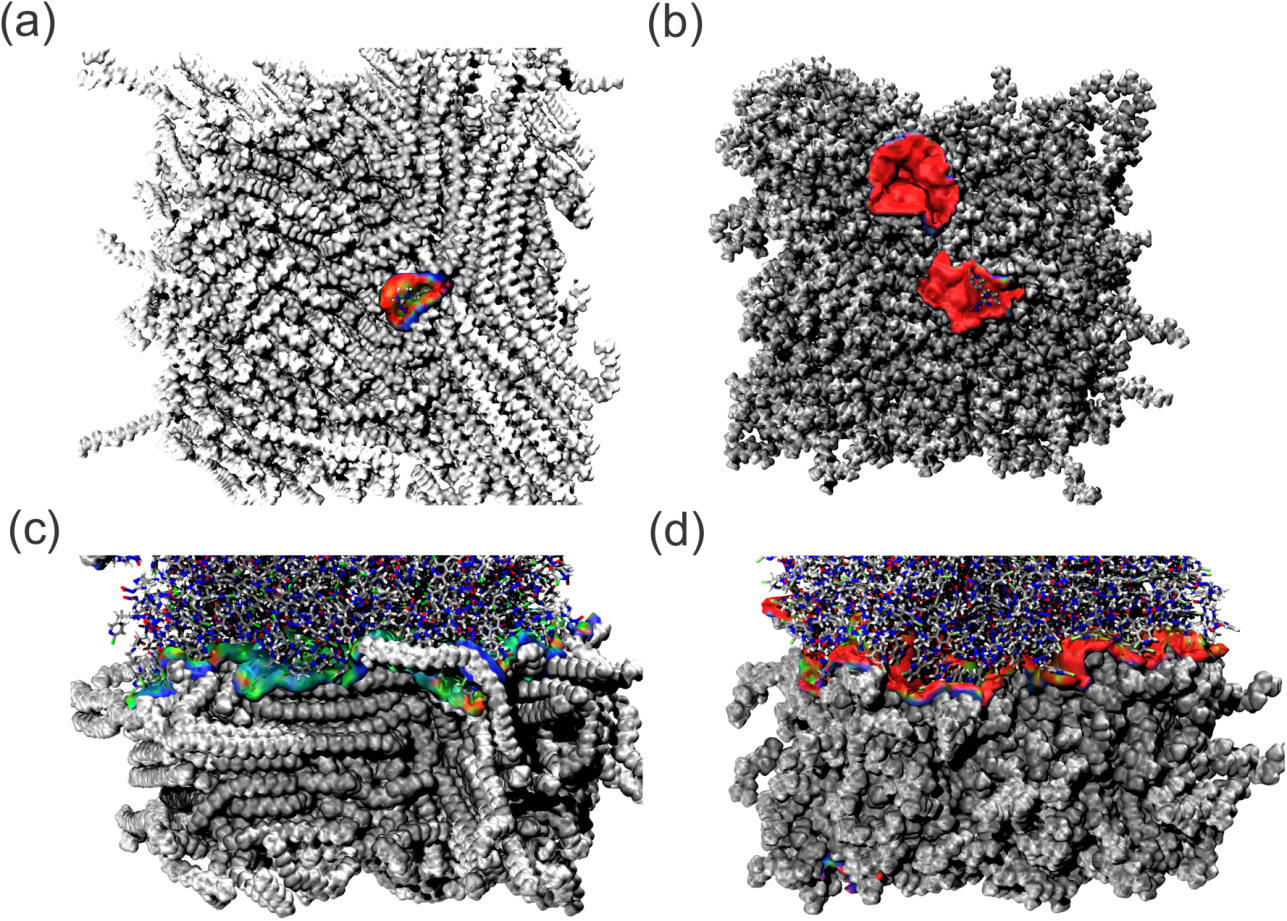
TFI isosurface map of IMI–MPs interactions of the (a) PE diluted, (b) PP diluted, (c) PE pure, and (d) PP pure systems.

The TFI index was utilized to assess the stability of the IMI–MPs interaction throughout the MD. Figure 7 illustrates that the PE systems’ interaction exhibited greater stability than the PP systems. This observation is evidenced by the predominance of green and blue shades in the PE systems, while the PP systems predominantly displayed red. This observation further supports the hypothesis that the imidacloprid molecule exhibits prolonged adsorption within a singular PE region. This finding is corroborated by the RDF, ROG, SASA, and IPE data.

### 3.3 Density Functional Theory

#### 3.3.1 Interaction Energy and Solvation Energy

From the DFT calculations, we obtained the interaction energies of each system. The energies were evaluated through electronic energy. The IMI diluted and pure systems were evaluated in this study to check for possible interactions with the PE and PP microplastics. From the DFT calculations, we obtained the interaction energies of each system. The adsorption energy with higher negative values is more favorable for the reaction, thus inducing stable MP–pesticide complex formation. According to our results, the pesticides studied interact more favorably with PE MP, with adsorption energies of −14.47 kcal mol^–1^. On the other hand, the interaction of these pesticides with PP MP was very similar, with interaction energies of −14.37 kcal mol^–1^. The solvation and intermolecular interactions of the complex, as well as its respective fragments, were evaluated. For this purpose, eq 9 was used to calculate the solvation energy.
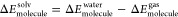
9
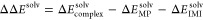
10

As shown in Table 1, the solvation energy is more favorable for the IMI molecule than for the MPs. The value of ΔΔ*E*^sol^ was obtained in eq 10, and the data analysis shows that the negative values (−2.81 and −1.97 kcal mol^–1^) reflect the influence of water effects on intermolecular bonding, indicating that the presence of the solvent stabilized the interaction energy. The observed phenomenon can be attributed to the presence of the polarity and partial charges of the IMI molecule on the surface. This is significant because MP alone exhibits no substantial polarity, and solvation in water is not favored, as evidenced by the positive of  values (5.80 and 10.48 kcal mol^–1^). Hence, when IMI interacts with the MP, it polarizes the surface and influences favorable solvation energy in the form of complexes. It can thus be concluded that solvation plays a significant role in the adsorption of IMI on the PE/PP surface and that it may act as a facilitating mechanism in the transport of IMI in aqueous media.

**Table 1:** Solvate Energies in kcal/mol^–1^

	Complex	MP	IMI	ΔΔ*E*^sol^
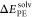	–13.58	5.80	–16.57	–2.81
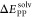	–9.42	10.48	–17.94	–1.97

Energy decomposition analysis based on force field analysis (EDA-FF) is used to study the strength of interaction between molecules to clarify the electronic nature of these energy differences. EDA-FF is based on the Amber force field, which decomposes the IMI–MP complex into two fragments, and the charges of the atoms of each fragment are calculated based on the Merz–Kollman charge. MPs generally have negligible polarity, but imidacloprid, in particular, has an average polarity of 9.569 debyes, which is considered high. Therefore, the interaction energy decomposition is fundamental to understanding the contribution ratios of total energy from the complex (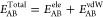
11

The Lennard–Jones potential of the interaction energy between atoms A and B is given by the sum of the repulsive energy , which represents the repulsion exchange interaction or Pauli effect and the interaction dispersion energy  (eqs 12 and 13). Thus, the contribution energy from atom A of the fragments is expressed in eq 11.
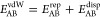
12
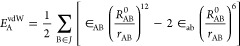
13

In the IMI–PE interaction,  was −1.50 kJ mol^–1^,  was 12.10 kJ mol^–1^, and  was −56.00 kJ mol ^-1^. With the sum, the value  was obtained. The IMI–PP interaction resulted in  , , and  , generating a  . Thus, the interaction between IMI–MPs is dominated by the dispersion energy, with significant contributions to the total binding energy. In contrast, the repulsive energy contributed significantly to both complexes. In a comparative analysis, the IMI–PE complex performed better in terms of interaction energy, as it obtained a lower , with around 1.5 kJ mol^–1^ difference, and a lower , with 2.9 kJ mol^–1^ less than the IMI–PP system. This difference can be attributed to the morphology of the polymer structures, since PE has a more distributed structure due to its CH_2_ monomer. At the same time, in the interaction with PP, IMI interacts mainly with the CH_3_ groups of propane.

#### 3.3.2 QTAIM

**Figure 8:**
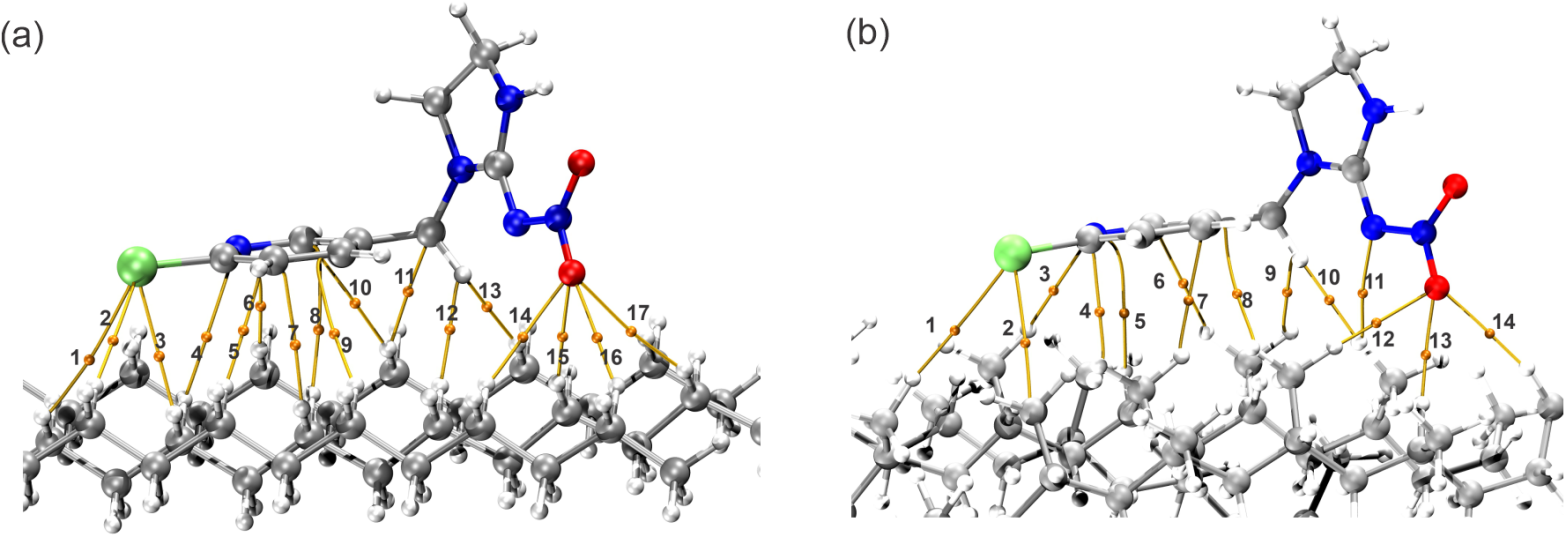
Molecular graphs of BCPs and path of bonds of the studied system IMI–PE (a) and IMI–PP (b).

The quantum theory of atoms in molecules (QTAIM) study of the geometrical structures of the IMI–MP complexes was carried out. Figure 8 shows the binding paths (yellow lines) and bond critical points (BCPs) related to the intersection of the electronic density of the interaction between the pesticide and the MP. The electron density in the BCP constitutes an aspect of utmost importance for understanding intermolecular interactions. On the PE surface, there were 17 interactions in which the chlorine and oxygen atoms stood out, with the highest electron density records (Table 2). However, there were fewer bonding paths and BCPs on the PP surface. It was also observed that the number of oxygen and chlorine interactions decreased by 1.

**Table 2:** Electron Density and Laplacian Spectra of the Electron Density of the IMI–PE and IMI–PP Complexes. Units in Eh

BCP PE	ρ	∇^2^ρ	BCP PP	ρ	∇^2^ρ
1	0.0023	0.0094	1	0.0023	0.0087
2	0.0060	0.0182	2	0.0030	0.0119
3	0.0066	0.0226	3	0.0077	0.0209
4	0.0033	0.0115	4	0.0082	0.0285
5	0.0051	0.0193	5	0.0032	0.0113
6	0.0060	0.0193	6	0.0035	0.0134
7	0.0049	0.0146	7	0.0062	0.0195
8	0.0044	0.0134	8	0.0026	0.0094
9	0.0023	0.0072	9	0.0029	0.0124
10	0.0043	0.0143	10	0.0075	0.0224
11	0.0041	0.0170	11	0.0042	0.0144
12	0.0071	0.0246	12	0.0094	0.0270
13	0.0040	0.0153	13	0.0093	0.0310
14	0.0054	0.0193	14	0.0081	0.0261
15	0.0065	0.0213			
16	0.0079	0.0289			
17	0.0025	0.0103			

In addition, it was observed that the hydrogen of the IMI methylene group has a significant interaction contribution, as evidenced by the electronic density values (ρ) of 0.0071 Eh (BCP 12) and 0.0075 Eh (BCP 10) for the PE and PP systems, respectively. Furthermore, the Laplacian of the electron density (∇^2^ρ) values is positive, indicating weak interactions. In addition, these values agree with the electronic density data, with the chlorine (BCP PE 1-3; BCP PP 1-2), hydrogen atoms of the methylene (BCP PE 12-13; BCP PP 9-10) and oxygen (BCP PE 14-17; BCP PP 12-14) and standing out.

#### 3.3.3 IGMH Study

**Figure 9:**
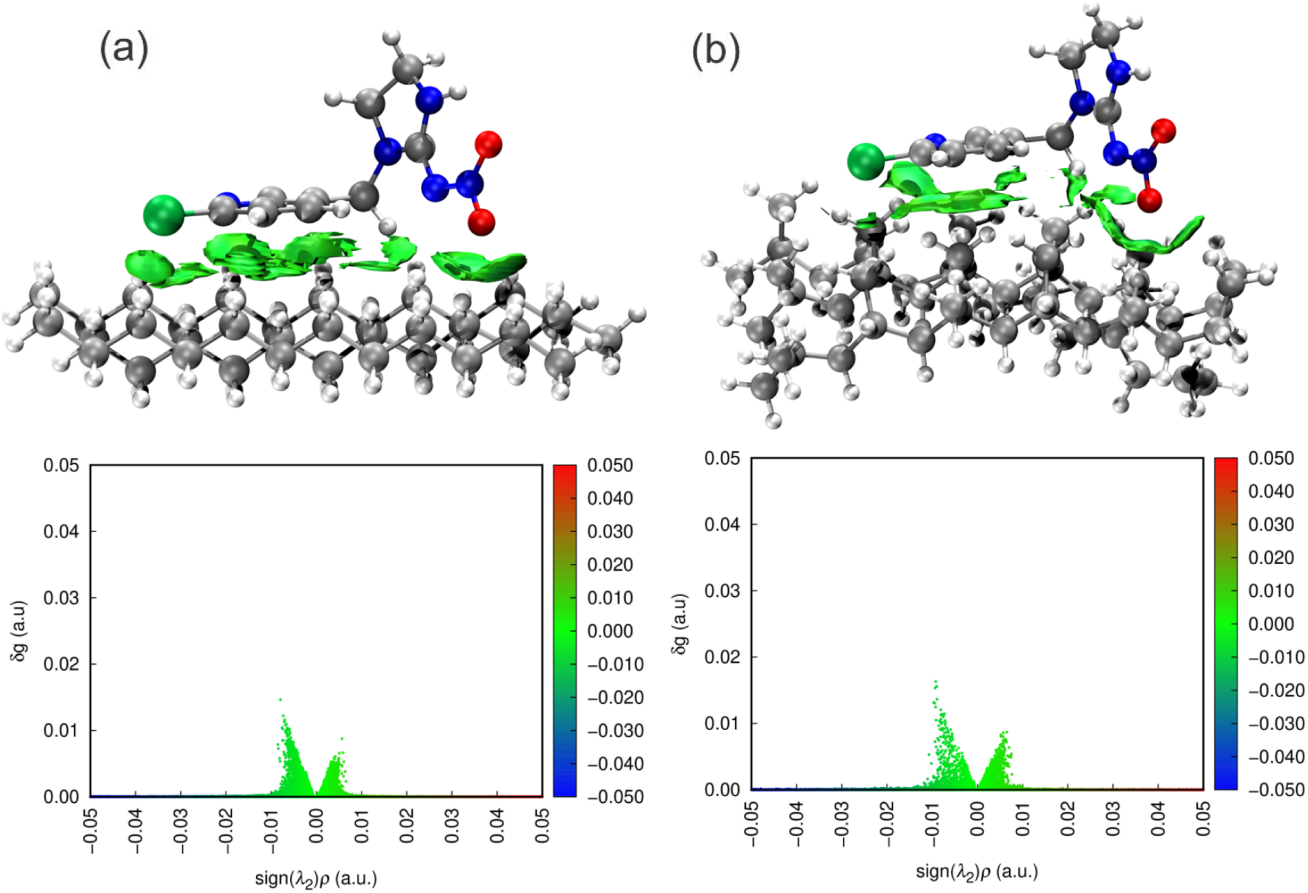
*g*^inter^ isosurfaces and RDG function versus sign (λ_2_)ρ of IGMH for the studied IMI–PE (a) and IMI–PP (b) complexes.

The IGMH figure indicates the noncovalent interaction potential of the systems studied. This map ranges from blue, indicating strong interactions such as hydrogen bonding, to green, indicating a medium interaction, such as van der Waals, to red, indicating a repulsive force. The graph of *g*^inter^ function versus sign (λ_2_)ρ also shows considerable interactions of an attractive nature. This is evidenced by the peaks in the 0.01 sign (λ_2_)ρ range for PE and PP, which are larger and denser than those in the negative sign (λ_2_)ρ, indicating that these interactions are more attractive than repulsive. However, the analysis was restricted to two fragments, namely the atom corresponding to imidacloprid and the MPs, analyzing only the intermolecular interactions between the surface and the pesticide. According to the image in Figure 3a, the graph of the *g*^inter^ function versus the (λ_2_)ρ signal indicates attractive interactions governed by van der Waals forces. For the IMI system, it indicates medium force interactions, which is evident in the graph of the *g*^inter^ function versus the sign ((λ_2_)ρ, which shows only the intermolecular bond between the oxygen and hydrogen of PE and PP, from the RDG isosurface map (Figure 9). The conformation for interaction with the surface favored the pyridine ring being perpendicular enough for van der Waals interactions, thus considering the more pronounced contribution of chlorine on the surface of PE, with a distance of 2.97 Å, compared to PP, with 3.09 Å. The nitro group of IMI favored the conformation perpendicular to the surface, interacting with a medium force through oxygen at a distance of 2.50 Å, thus maintaining a resonance balance of the nitro group aided by intramolecular hydrogen bonding with the imidazole ring. The same trend is observed for interactions with the PP surface with a relatively shorter distance (2.45 Å), increasing the intensity of the interaction.

#### 3.3.4 Frontier Orbitals (FMO)

**Figure 10:**
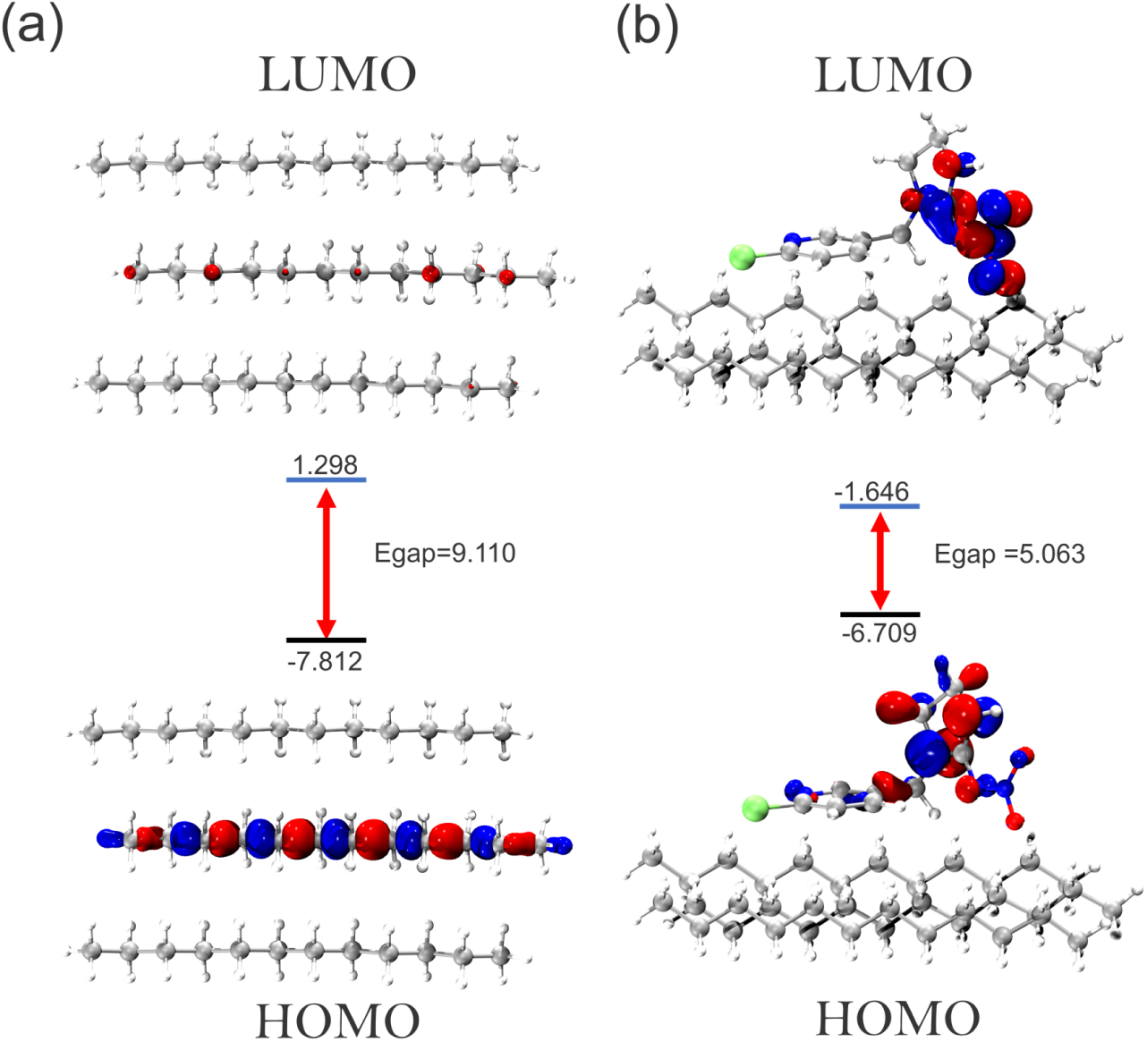
Distributions and parameters of HOMO and LUMO orbitals for the PE surface (a) and IMI–PE complex (B). The energy is in electronvolt (eV).

**Figure 11:**
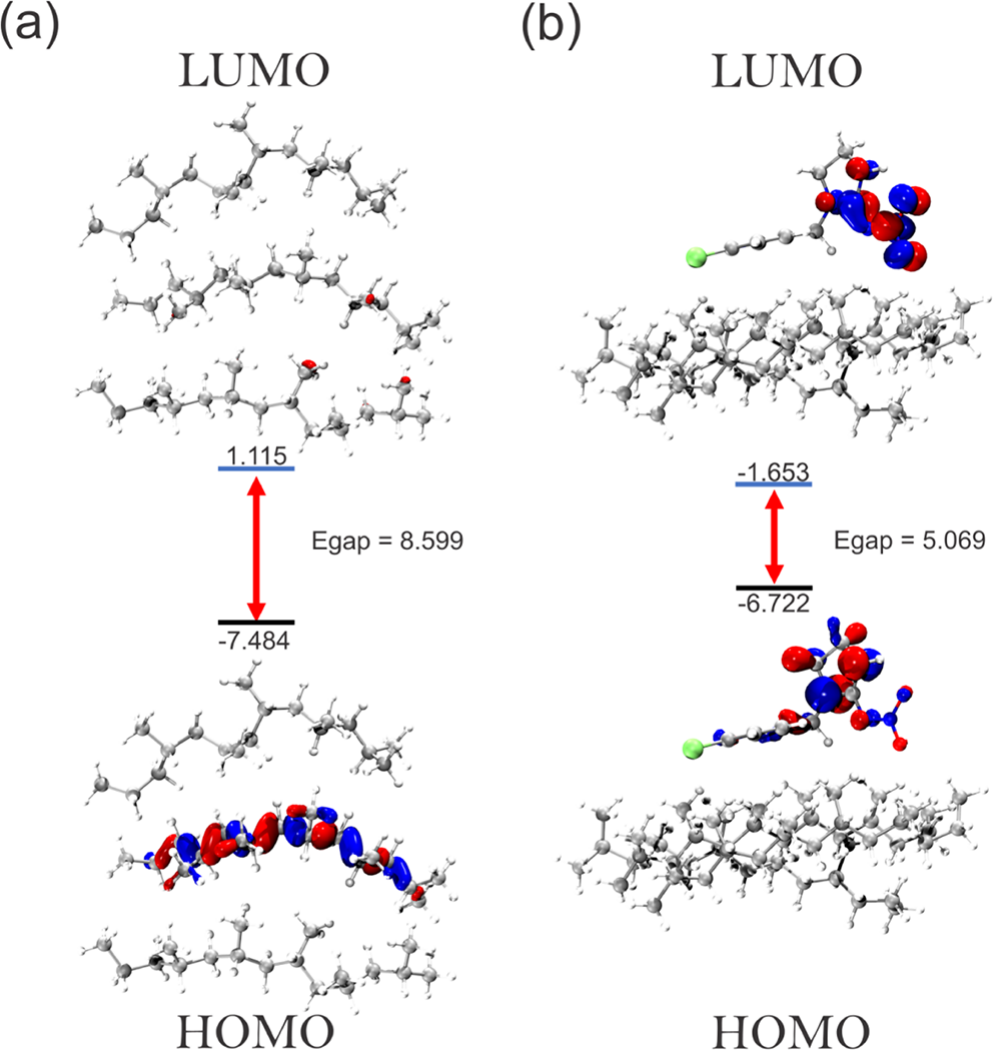
Distributions and parameters of HOMO and LUMO orbitals for the PP surface (a) and IMI–PP complex (b). The energy gap (*E*_gap_) is in electronvolt (eV).

Figures 10 and 11 show the HOMO (highest occupied molecular orbital) and LUMO (lowest unoccupied molecular orbital) frontier orbitals (FMO), comparing the MP surface and its respective adsorbed complexes. The gap energy (*E*_gap_) indicates the chemical stability of the system, it is calculated by the difference in the energies of the frontier orbitals (*E*_homo_ and *E*_lumo_). It can be seen that the PE surface has a larger *E*_gap_ compared to PP, with values of 9.110 and 8.599 eV, respectively. Thus, both surfaces indicated a high reactive stability from the FMO. However, after IMI interacts with the surface, the energy gap *E*_gap_ values are very similar, with values of 5.063 and 5.069 eV for PE–IMI and PP–IMI, respectively. The similar *E*_gap_ values corroborate the results of the electronic interaction energy, indicating that the energy of the frontier orbitals of the IMI–MPs complex significantly impacts understanding the interaction energy. The variation in the surface *E*_gap_ for the MP–IMI complex was −4.047 and −3.530 eV for the PE and PP systems, respectively. With these values, it can be seen that the fact that *E*_gap_ decreased after IMI adsorption indicates a lower chemical stability in the MP–IMI complex, which could be an indication of the nature of the weak force interaction. In addition, the IMI–MP interaction, although favorable and consistent with the interaction energy data via DFT and IPE MD, may suggest that it does not have sufficient chemical stability to be considered to be chemisorption. This type of interaction could be a risk, as the MP–IMI could be favorable enough for a possible IMI transport vehicle and gradually being released by the indicative of a physisorption nature.

#### 3.3.5 Density of States (DOS)

**Figure 12:**
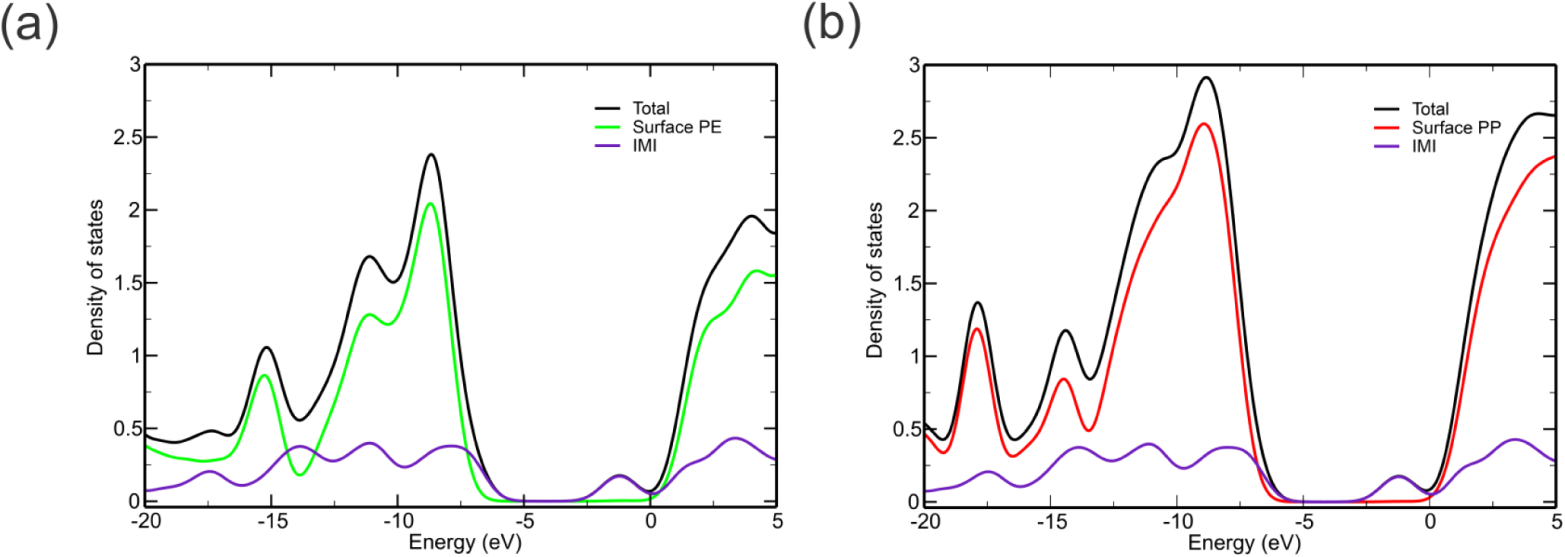
DOS spectra of complexes IMI–PE (a), IMI–PP (b).

To determine the density of states (DOS) for a system composed of an adsorbent and an adsorbate using density functional theory (DFT), it is necessary to obtain the electronic band structure of the combined system initially. This can be done by conducting DFT calculations on the adsorbent and adsorbate separately and on the combined adsorbate–adsorbent system. The electronic structures of the isolated and combined systems can then be compared to provide insight into the electronic interactions between the adsorbate and adsorbent. The DOS can be calculated for both the adsorbent and the adsorbate individually and for the combined system. By analyzing and comparing the adsorbent and adsorbate DOS with that of the combined system, one can determine the changes in the electronic structure that occur during adsorption. Figure 12 shows that the HOMO states are slightly higher in energy when the pesticide is adsorbed on the PE surface. The LUMO states of the pesticides did not change significantly for IMI. In addition, we can observe the contributions of the electronic density populations of the pesticides (blue line) and compare this contribution to the adsorbate–adsorbent system. Comparing the contributions of the pesticides, IMI has characteristics that contribute to the electronic population of the complex, thus corroborating the favorable adsorption energy and MD and DFT results for the PE and PP surfaces. Both graphs show the superposition of the IMI orbits and the surfaces for the formation of the complex. In parallel, the LUMO energy of IMI was shifted to the right in both systems, demonstrating that the MP surface favored the availability of densities of states and the stabilization of the IMI–MPs complexes formed.

## 4 Conclusions

The nature of the interaction between the pesticide imidacloprid and the PE and PP surfaces, as an adsorption phenomenon, was characterized by MD and DFT analyses. The interaction energy values for the adsorption of IMI molecules on the PE surface are higher in pure, although there is no significant difference in dilute solutions. The DFT study indicates that the interaction is favorable and that solvation favors the formation of the MP–IMI complex. In summary, this study provides a molecular understanding of the interaction between MPs and IMIs, demonstrating that the interaction is exothermic and governed by van der Waals forces. Consequently, this study is a precursor aiming to observe and predict the possibility of IMI adsorbing to MP, which is a possible carrier of this pesticide in the environment. MP functions as a direct vector for the release and dissemination of the pesticide, which represents a risk to the environment and also considers the risks of IMI to human health. Therefore, this study encourages experimental analyses of adsorption kinetics and isotherms, as well as the proposal of the respective models, in order to analyze and prove the adsorption behavior of IMI in PE and PP.
